# Givers and Giving

**Published:** 1888-12-22

**Authors:** 


### The Hospital

A Weekly Institutional Journal of

**Science, Medicine, Nursing, and Philanthropy.**

*For Hospitals, Asylums, Medical Practitioners, Students, Nurses, Families, Parishes, Congregations, and the Charitable Public.*

Vol. V. No. 117.] [December 22, 1888.

### Givers and Giving.

The small creatures that build up coral reefs accomplish their task very slowly, and each minute organism contributes an infinitesimal quantity to the result. But coral reefs are built up, and stand boldly out of the waters of the ocean, monuments of noiseless, deliberate, but effective power. An impatient observer, watching the process from day to day, might deny that progress is being made, and scout the idea that any completed result will ever demonstrate itself to the sight. Similarly, benevolence and generosity were, in the early ages of the world, feeble in intensity and limited to a few individuals. In the nature of things it could not be otherwise. Human beings, born into the environment of a fierce struggle for existence, were compelled to live as they could, if not always as they would. But the few who were inspired with the noble ideas of benevolence and generosity had their place in the scheme of things as definitely ordered as the reef-builders. They were not conscious of the great part they had to play in the development of intellect, conscience, and benevolence, any more than the reef-builders are conscious of the island which may be the final result of their united operations. But as surely as coral reefs have arisen from the depths of the sea, and proclaim themselves not only to their own inhabitants, but to every passing ship, so surely does the world of benevolence proclaim its bold and striking concrete existence to every man in these islands who has eyes to see. There has emerged during the ages from the seeming chaos of "life struggles" and "survivals of the fittest," an ordered and sure world of benevolence, generosity, and duty-loving regard for persons and things outside and away from self. The result is at least striking.

It would not be unprofitable, from a science-of-man point of view, to make some strictly scientific inquiries on the lines here suggested. The facts are undoubtedly as we have stated them; and science is nothing if she does not take account of facts, and less than nothing if she cannot offer some explanation of them which shall be at least plausible. But our purpose at present is practical, not academic. The world of benevolence is here. We will glance at it for a brief period as it is, and also consider what it might be. The man who does not admire the work done by benevolent and generous persons in this country as it now is, must be a stupid churl. Undoubtedly the noble motives, the self-denial, the energy, and the conscientiousness displayed by those—and they are many—who devote their lives and fortunes to philanthropy, are worthy of deep admiration. Those who sneer at philanthropists, because they sometimes do impulsive things which are not justified by results, do not thereby prove their own superior intelligence. Money-making men of business, and self-admiring men of the world, have been occasionally known to do things which even less clear-sighted men than Solomon would not have denominated "wise." There are those who whisper a suspicion that men who give themselves exclusively to money-making or pleasure-seeking, are sometimes—to use one of their own phrases—"very much out of it" when taken away from their own peculiar beat. Nay, more, it is hinted that they are not always "in it" even on their own beat. It is not improbable that if a candid and able sociologist were asked which of the three classes—philanthropists, money makers, or pleasure-seeking men of the world—was highest in the region of pure "brains," he would give the first place to the philanthropists. Pure philanthropy undoubtedly develops the intellect; mere money-making as surely develops the foxy or weasel-like qualities, and mere pleasure-seeking as unmistakeably suggests the characteristics of the Christmas animal who lives in a "sty."

Philanthropists as they are deserve well of the community; as they might be they would deserve monuments of marble and of gold. The one thing many generous people lack is what is called the "scientific method." Let nobody be frightened by the phrase. The scientific method has no mystery about it; the essence of it is this—to see the facts as they are, and to deal with them as they are. But though there is no mystery, there is great difficulty. People whose work in life is the noble business of philanthrophy generally have a method of some kind. Their operations are so numerous, varied, and extensive, that necessity compels them to a method. Without it their work would come to a dead-lock. Of course, every philanthropist has not the same method. A good many, however, and those of the noblest class, have stereotyped rules, which much diminish the value of their work. They are like gas-fitters or water-pipe engineers. They resolve that certain things are to be done in certain towns and neighbourhoods at certain times. They keep their reservoirs of ready money, blankets, coals, hospital tickets, and so on always well filled; they lay on their pipes to the different objects of their bounty; they fix the taps and set their engines in order, and then they "play away."

Now, that method is good provided it is not used rigidly or exclusively. Water-pipes are very necessary, but sometimes they get out of order; tenants will leave, and so water may be wasted; circumstances change, and ten or a hundred times as much water is imperatively required at a given point, and cannot be waited for without a terrible catastrophe. Yet many philanthropists—most excellent persons—will not brook any suggestions of possible change; they will not even look at schemes outside their "water-pipe" system. Their plans are made, their money is allocated for the year, their consciences are satisfied, and they fold their hands with a not unjustifiable content. But no affairs which Providence allots to man are of this rigid and mechanical kind. It is clearly the intention of the Ruler of the world that a good deed shall bless "him that gives as well as him that takes." But the giver can only be fully blessed on one condition, and the condition is that the whole of his intellect and conscience shall be put into each separate act of benevolence.

Giving must be intelligently and scientifically done. If it fails here, it may, and often does, fail entirely. Not only does it fall short of the full good contemplated, but it very frequently does an infinity of harm. Givers must not think that no responsibility attaches to them for the harm done. They are apt to consider that because the motive was good the mischief resulting from their act cannot be charged upon them. It both can be and ought to be charged upon them. Men and women have intelligence as well as kindly feelings. Intelligence is a higher endowment than kindly feeling, and involves a correspondingly higher responsibility. A horse or a dog may be as kind and as faithful as a man; he cannot be so intelligent. Will anyone charge a dog with the same responsibility as a man? It devolves upon all philanthropists, therefore, to bring the same amount of intelligence and thoroughness to their work as they would if they were making scientific investigations or conducting a private business in which large sums of money were involved. This implies two conditions: a clear understanding of every question at the outset, and an unremitting watchfulness. Every institution which they help with their money should be inspected thoroughly, and all its proceedings periodically revised. Instead of a "water-pipe" system of mechanical philanthropy, there should be an active business, conducted by living, intelligent, watchful and unprejudiced men and women, who will give to every new claim the consideration it deserves, and to every worn-out plea the prompt *coup de grace*. This of course means work, discretion, intellect, energy, all the qualities that go to the making of men and women of the highest intellectual and moral stamp. But it means also the highest rewards which Time has for her children, and the infinitely noble hopes of the future that lies beyond. The mightiest impulse ever brought to bear upon intelligent benevolence and self-sacrifice was given by Him whose name and birth we celebrate at this Christmas season. He who would devote his life to the noblest of all human purposes, will nowhere find such inspiration and potent energy as in Jesus Christ of Nazareth.

